# dRFEtools: dynamic recursive feature elimination for omics

**DOI:** 10.1093/bioinformatics/btad513

**Published:** 2023-08-26

**Authors:** Kynon J M Benjamin, Tarun Katipalli, Apuã C M Paquola

**Affiliations:** Lieber Institute for Brain Development, Baltimore, MD 21205, United States; Department of Neurology, Johns Hopkins University School of Medicine, Baltimore, MD 21205, United States; Lieber Institute for Brain Development, Baltimore, MD 21205, United States; Lieber Institute for Brain Development, Baltimore, MD 21205, United States; Department of Neurology, Johns Hopkins University School of Medicine, Baltimore, MD 21205, United States

## Abstract

**Motivation:**

Advances in technology have generated larger omics datasets with potential applications for machine learning. In many datasets, however, cost and limited sample availability result in an excessively higher number of features as compared to observations. Moreover, biological processes are associated with networks of core and peripheral genes, while traditional feature selection approaches capture only core genes.

**Results:**

To overcome these limitations, we present dRFEtools that implements dynamic recursive feature elimination (RFE), reducing computational time with high accuracy compared to standard RFE, expanding dynamic RFE to regression algorithms, and outputting the subsets of features that hold predictive power with and without peripheral features. dRFEtools integrates with scikit-learn (the popular Python machine learning platform) and thus provides new opportunities for dynamic RFE in large-scale omics data while enhancing its interpretability.

**Availability and implementation:**

dRFEtools is freely available on PyPI at https://pypi.org/project/drfetools/ or on GitHub https://github.com/LieberInstitute/dRFEtools, implemented in Python 3, and supported on Linux, Windows, and Mac OS.

## 1 Introduction

**Figure 1.**
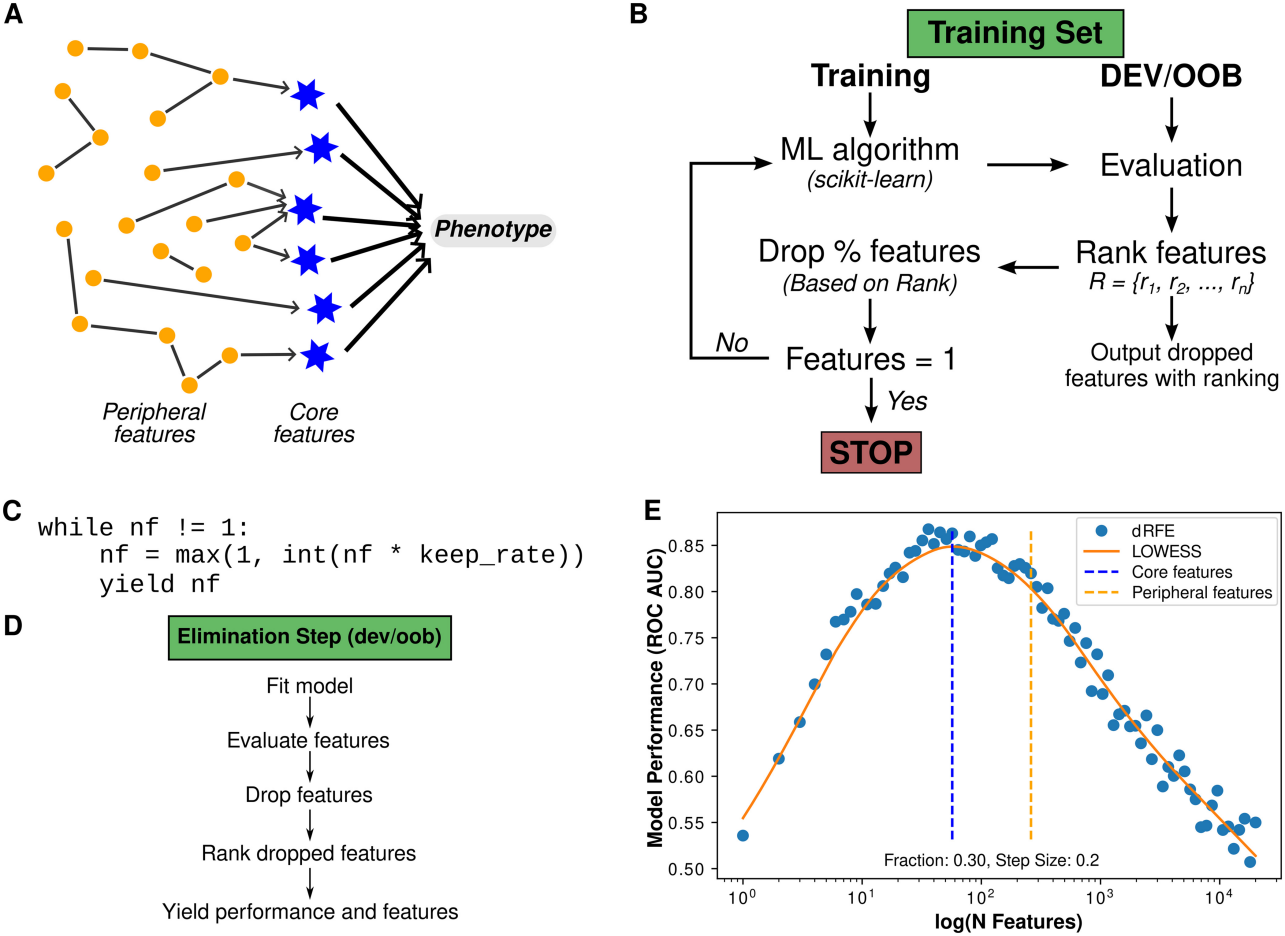
Schematic of dynamic recursive feature elimination for dRFEtools. (A) Graphical representation of core and peripheral features (Boyle et al., 2017). (B) Flowchart showing recursive elimination process, where scikit-learn model can be either classification or regression. We ranked the dropped features and saved them for downstream analysis. (C) Feature iterator code used to generate dynamic elimination. (D) Flowchart showing the elimination steps using the developmental or out-of-bag (OOB) set. (E) Example of LOWESS fitting on dRFEtools model performance multiple classification using simulated data using area under the receiver operating characteristic curve (ROC AUC).

The creation of increasingly larger epigenetics, genetics, and transcriptomic datasets from high-throughput sequencing has generated more comprehensive insights into human biology [e.g. identification of biomarkers and novel therapeutics for various diseases (Matthews et al., 2016, Perakakis *et al.* 2018)]. However, costs and limited sample availability result in an excessively higher number of features compared to observations. This can result in overfitting, which feature selection approaches can solve. Moreover, biological processes are associated with networks (Boyle et al., 2017) including core (direct, large effects) and peripheral (many small, indirect effects) genes (Fig. 1A). As such, it is often biologically relevant to select a subset of features that include core and peripheral genes. However, traditional feature selection approaches are optimized to select only core features, limiting biological interpretability.

Recursive feature elimination (RFE) is an iterative process that optimally removes one feature at a time. For computational considerations, we can eliminate a substantial number of features (feature subset ranking); however, it can be difficult to balance computational time (small number of features dropped) and model performance degradation (substantial number of features dropped). To overcome this issue, RFE can be done dynamically, which provides a more flexible feature elimination operation by removing a substantial number of features at the beginning and becoming a single feature elimination when there are a small number of features. Here, we present dRFEtools—a Python package that integrates with scikit-learn and implements dynamic RFE (Nguyen and Ohn 2006). In addition to reducing computational time with high prediction accuracy, dRFEtools expands dynamic RFE to regression problems and outputs subsets of features that hold predictive power with and without peripheral genes.

## 2 Implementation

The purpose of dRFEtools is to implement dynamic RFE for classification and regression using the available supervised learning models on scikit-learn with coef_ or feature_importances_ attribute (e.g. decision trees and linear models). dRFEtools provides fast and accurate feature selection as compared to standard RFE, which is either fast (large step size) or accurate (small step size) for big omics datasets (i.e. features > 20 000). The development of dynamic RFE (Fig. 1B) for scikit-learn models can be separated into two main parts: (i) feature iterator (Fig. 1C) and (ii) model evaluation for *N* features (Fig. 1D). We use the feature iterator to control the dynamic selection of features to eliminate as opposed to removing a static step size, as is done in traditional RFE. For interpretability, we rank all features.

For each iteration, we evaluate feature importance (i.e. absolute weights or unsigned Gini) using a random validation set to reduce the chance of overfitting. To evaluate model performance during dynamic RFE, we assess classification accuracy (i.e. accuracy, AUC ROC, and normalized mutual information) or regression correlation (i.e. $r^{2}$, mean square error, and explained variance). We recommend using *n*-fold cross-validation with this method to further reduce the overall chance of overfitting.

From the locally weighted scatterplot smoothing (LOWESS) curve, we extract the local maximum as the core feature set (Fig. 1E). To extract core and peripheral features, we examine the rate of change of the LOWESS curve and select the point at which the slope changes steeply because we, at this point, assume additional features offer no contribution and reduce prediction accuracy. We provide two functions to extract the core (extract_max_lowess) and core+peripheral (extract_peripheral_lowess) features to be used with main dynamic RFE functions (rf_rfe or dev_rfe). The main functions return a dictionary with all dynamic RFE results that we use to extract predictive features for downstream test set evaluation. An example of optimization and classification codes are available at https://pypi.org/project/drfetools/.

## 3 Application and validation

To assess the ability of dRFEtools to accurately identify informative features in classification and regression problems, we performed two different simulation analyses (scikit learn- and omics-based) to compare dRFEtools with the current RFE scikit-learn function. We used eight popular algorithms: four for classification (logistic regression, random forest classifier, stochastic gradient descent, and support vector classification) and four for regression (ridge, elastic net, random forest regressor, and support vector regression). We applied these algorithms on the test set to measure: (i) feature selection accuracy, (ii) feature selection false discovery rate (FDR), and (iii) computational time. For our biological simulation, we simulated bulk RNA-sequencing and quantitative trait loci (QTL). We found that computational time and FDR of informative features were significantly reduced (dRFEtools versus RFE) in both classification and regression models (one-way ANOVA, *P*-value < 0.01; Supplementary Figs S1–S4).

To illustrate dRFEtools application to biological data, we considered a subset of data from the BrainSeq Consortium Phase 1 DLPFC adult (age > 17) postmortem brain collection (*n* = 521) (Jaffe *et al.* 2018). With this dataset, we considered three scenarios: (i) binary classification for schizophrenia (*n* = 172) and major depression disorder (MDD; *n* = 142) using gene expression from poly-adenylated RNA-sequencing as features, (ii) multi-class classification of neuropsychiatric disorders (neurotypical control, *n* = 207) using gene expression as features, and (iii) regression modeling to impute gene expression using SNP genotypes as features. We found dRFEtools provided biological relevant core and peripheral features applicable for pathway enrichment analysis and expression QTL (Supplementary Figs S5–S8).

## Supplementary Material

btad513_Supplementary_Data

## Data Availability

The hg38-aligned gene expression datasets analyzed in the current study are available upon request. The original hg19-aligned R variables are available at http://eqtl.brainseq.org/phase1/. The FASTQ files for all BrainSeq Phase 1 subjects (*n* = 738) are available on Synapse (Collado-Torres *et al.* 2019). Genotypes are available from Globus with restricted access (Collado-Torres *et al.* 2019). More information on the BrainSeq publicly available data can be found at http://eqtl.brainseq.org/. dRFEtools is available on Python Package Index (PyPI) at https://pypi.org/project/drfetools/ and on GitHub at https://github.com/LieberInstitute/dRFEtools. The code and Jupyter notebooks that produced the results for this manuscript are available through GitHub at https://github.com/LieberInstitute/dRFEtools_manuscript.
